# Comprehensive characterization of FBXW7 mutational and clinicopathological profiles in human colorectal cancers

**DOI:** 10.3389/fonc.2023.1154432

**Published:** 2023-03-29

**Authors:** Yiping Liu, Hanlin Chen, Hua Bao, Jinfeng Zhang, Runda Wu, Lingjun Zhu

**Affiliations:** ^1^ Department of Oncology, Xiangya Hospital, Central South University, Changsha, China; ^2^ Geneseeq Research Institute, Nanjing Geneseeq Technology Inc., Nanjing, China; ^3^ Department of General Surgery, The First Affiliated Hospital of Soochow University, Suzhou, China; ^4^ Department of Oncology, The First Affiliated Hospital of Nanjing Medical University, Nanjing, China

**Keywords:** Fbxw7, colorectal cancer, next generation sequencing - NGS, biomarker, GSEA analysis

## Abstract

**Background:**

FBXW7 is recognized as a critical tumor suppressor gene and a component of the ubiquitin-proteasome system, mediating the degradation of multiple oncogenic proteins, including c-MYC, Cyclin E, c-Jun, Notch, p53. Around 16% of colorectal cancer (CRC) patients carried FBXW7 somatic mutations, while a comprehensive characterization of FBXW7 somatic mutations in CRC is still lacking.

**Methods:**

Colorectal cancer patients with tumor samples and matching white blood cell samples in the past five years were screened and DNA sequenced. DNA sequencing data of MSK MetTropism cohort and RNA sequencing data of TCGA COAD cohort were analyzed.

**Results:**

We discovered that the FBXW7 mutations were associated with higher tumor mutation burden (TMB), higher microsatellite instability (MSI) score, and lower chromosomal instability (CIN) score. Patients with FBXW7 mutations showed better overall survival (HR: 0.67; 95%CI: 0.55-0.80, P < 0.001). However, patients with FBXW7 R465C mutation displayed worse overall survival in multi-variate cox analysis when compared with patients carrying other FBXW7 mutations (HR: 1.6; 95%CI: 1.13-3.1, P = 0.015), and with all other patients (HR: 1.87; 95%CI: 0.99-2.5, P = 0.053). Moreover, in MSI patients, the FBXW7 mutated group showed higher M1 macrophage, CD8+ T cell, and regulatory T cell (Tregs) infiltration rates, and significant enrichment of multiple immune-related gene sets, including interferon-gamma response, interferon-alpha response, IL6 JAK STAT3 signaling, p53 pathway.

**Conclusion:**

This analysis comprehensively identified FBXW7 alterations in colorectal cancer patients and uncovered the molecular, clinicopathological, and immune-related patterns of FBXW7-altered CRC patients.

## Introduction

Colorectal cancer (CRC) was the third leading cause of cancer incidence and death in 2021, with an estimated over 150000 new cases and 52000 mortalities in 2022 (1). During the past decade, both the mortality and incidence rate of CRC dropped by around 2% each year due to the increasing early prevention by colonoscopy (1, 2). Five-year survival for metastatic disease stayed at 15%, calling for improving therapeutic choices (1). It is commonly acknowledged that the molecular subsetting of CRCs can facilitate the therapeutic decision-making for targeted agents and radiation, as patients with different molecular profiles present distinct clinical outcomes and treatment responses (3, 4). For instance, microsatellite instability-high (MSI-H) CRC patients achieved better responses toward immune checkpoint inhibitor therapy (5), and KRAS mutations were a negative predictor of response to panitumumab or cetuximab therapies, which both are first-line treatments of CRC (6). Therefore, studying the molecular profiles of CRC patients may provide hints for novel targeted therapeutical interventions and clinical outcome prediction.

FBXW7 (F-box/WD repeat domain-containing 7) was recorded in around 14% of CRC patients (7), as one of the most frequently mutated genes in CRC, along with APC, TP53, and KRAS (8). FBXW7 was recognized as a critical tumor suppressor gene and a part of the SCF (SKP1-CUL1-F-box protein) complex, an E3 ubiquitin ligase that conducts protein ubiquitylation and stimulates subsequential proteosome-mediated degradation (9). Furthermore, FBXW7 is known to be responsible for the degradation of multiple oncogenic genes, including c-MYC, Cyclin E, c-Jun, Notch, p53, and inactivation of it may disrupt the downstream signaling pathways, which can lead to respective disease conditions, especially tumorigenesis (10). FBXW7 was associated with the DNA damage response and repair process by regulating FA (Fanconi Anaemia) and NHEJ (non-homolog end-joining) pathways (11).

In previous research, the correlations between FBXW7 mutations and pathological characteristics in colorectal cancer were inconsistent. In a study involving 1519 colorectal cancer patients, FBXW7 mutations were more common in stage I/II patients (12), while a meta-analysis involving 4199 patients showed that FBXW7 mutations were correlated with advanced T stage and lymph node metastasis (13). Regarding other types of tumors, in gastric cancer, FBXW7 mutated patients had larger tumor size (14). In human breast cancer, ERBB2 and basal tumors showed significantly lower average FBXW7 expressions than normal-like tumors (15). Moreover, the clinical significance of FBXW7 mutations in colorectal cancer remains controversial. Multiple studies reported FBXW7 alterations as prognostic indicators in CRC, yet the results were not consistent among different studies (12, 16–18). Hence, a detailed and comprehensive study exploring the molecular and clinicopathological profiles of FBXW7 mutated CRC patients is still lacking.

In this series, three cohorts were analyzed for different aims. A dataset of 7626 CRC tumors was profiled to uncover the genetic and clinicopathological characteristics of FBXW7 mutated patients. Clinical outcomes and risk factors were analyzed using 3541 CRC patients from the MSK cohort with overall survival data. Immune cell infiltration and gene set enrichment analyses were conducted on RNA sequencing data from 263 patients in the TCGA COAD cohort.

## Materials and methods

### Study cohort

Tumors and matching white blood cell samples from 7638 CRC patients were sequenced by a panel targeting 425 cancer-related genes. Samples with no nonsynonymous mutation were excluded from the analyses. All patients/participants provided their written informed consent upon the sample collection data and the study was approved by the Ethical Committee of The First Affiliated Hospital of Nanjing Medical University (Approval No. 2022-SR-294).

**Figure 1:**
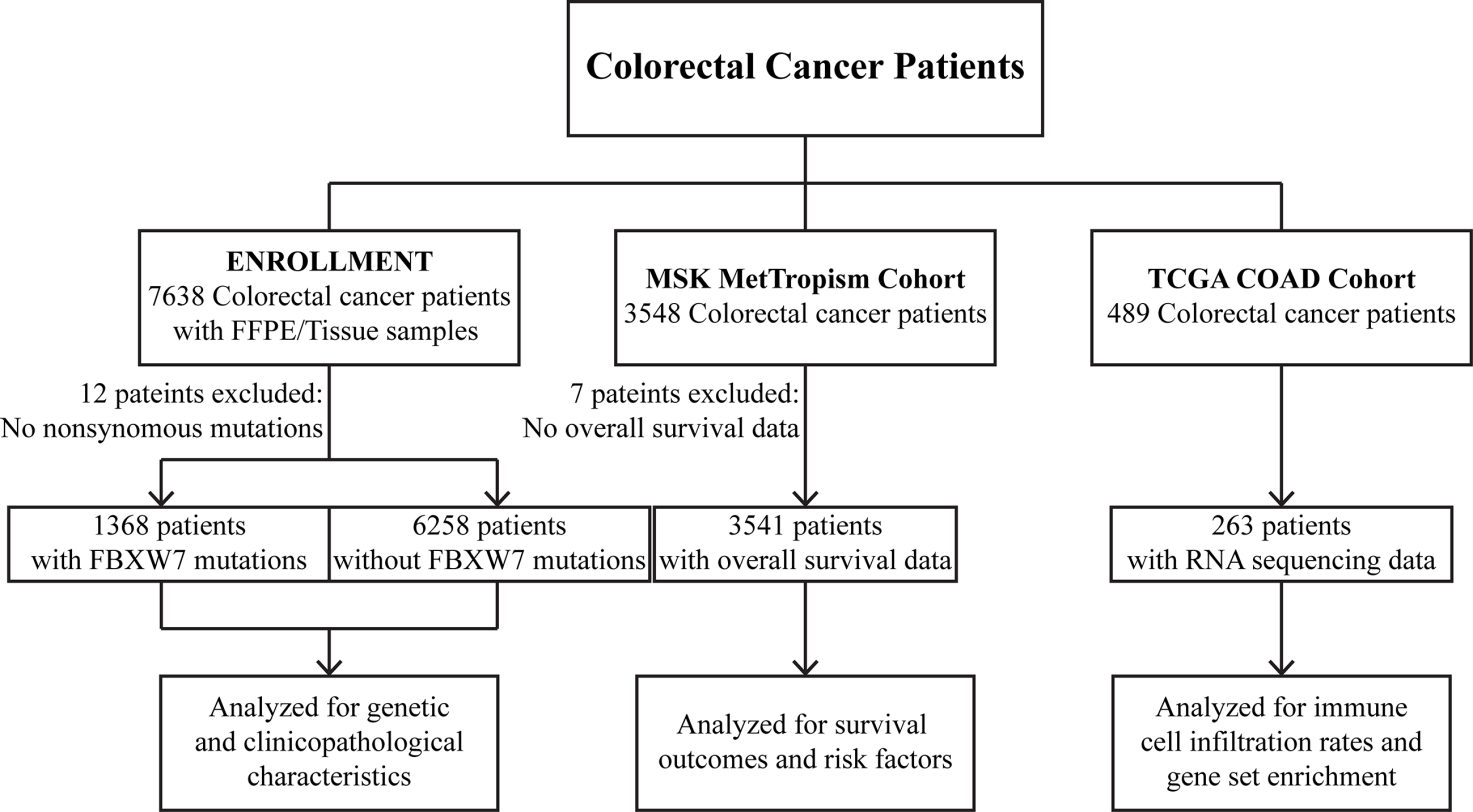
Flow chart of the study.

Two published independent data sets were also analyzed in this study. 3541 colorectal cancer patients with overall survival data in the MSK MetTropism (https://www.cbioportal.org/) cohort were analyzed for survival outcomes and risk factors. 263 patients with RNA sequencing data in the TCGA COAD (https://portal.gdc.cancer.gov/projects/TCGA-COAD) cohort were analyzed for immune cell infiltration rates and gene set enrichment. Detailed study design is shown in **Figure 1**.

### DNA extraction and sequencing library preparation

As previously described (19), genomic DNAs in formalin-fixed paraffin-embedded (FFPE) tissues and blood control samples were extracted with QIAamp DNA FFPE Tissue Kit and DNeasy Blood and Tissue Kit (Qiagen, Hilden, Germany). The extracted DNA was quantified and quality-assessed using the Qubit 3.0 fluorometer and Nanodrop 2000 (Thermo Fisher Scientific). KAPA Hyper Prep Kit (KAPA Biosystems) was applied for sequencing library preparation. The capture reaction was performed on Dynabeads M-270 (Life Technologies, Carlsbad, CA, USA) and xGen Lockdown; the amplification of the obtained libraries was processed using KAPA HiFi HotStart ReadyMix (KAPA Biosystems) on bead; purification was performed on Agencourt AMPure XP beads (Beckman Coulter). Captured libraries were quantified by qPCR with the KAPA Library Quantification Kit (KAPA Biosystems), and the fragment size was assessed by Bioanalyzer 2100 (Agilent Technologies, Santa Clara, CA, USA). The resulting library was sequenced using HiSeq4000 NGS platforms (Illumina, San Diego, CA, USA) following manual instructions.

### Next generation sequencing

Sequencing data were processed as previously described (19). In short, the data was demultiplexed, and “Trimmomatic” was applied to filter low-quality data (quality < 20) and N bases (20). The qualified reads were aligned to the reference human genome (Human Genome version 19) with “Burrows-Wheller Aligner”. “Genome Analysis Toolkit” (GATK 3.4.0) was used for local realignments around indels together with base score recalibration, and deduplication was performed on Picard. Mutation calling and annotation were performed using “VarScan2” and “vcf2maf”, respectively. Insertion and deletion mutations were called with “VarScan2”. Single-nucleotide variations (SNV) with a variant allele frequency (VAF) of less than 1% in tissue samples and 0.3% for plasma samples were removed from the analysis. SNVs labeled as presented in more than 1% population in the 1000 Genomes Project or the Exome Aggregation Consortium (ExAC) 65,000 exomes database were filtered. Sequencing artifacts, germline variants, and clonal hematopoiesis were removed by parallel sequencing of tumor-normal blood sample pairs. The tumor purities were estimated using “ABSOLUTE” and samples with a tumor purity larger than 0.2 were kept for further analysis (21).

### Microsatellite instability score, tumor mutation burden, hypermutation status, and chromosomal instability score definition

Microsatellite instability (MSI) was determined based on the status of 52 indel sites covered by the targeted NGS panel. The panel MSI status detection ability was validated using 90 samples with “promega MSI analysis system v1.2” as a reference. The panel displayed an accuracy of 95.6% (sensitivity: 96.8%; specificity: 94.9%). The cutoff was set as the Youden index of the ROC curve generated by the assay validation. Tumor mutation burden (TMB) was calculated as the total number of nonsynonymous somatic mutations per megabase (Muts/Mb). Patients with TMB higher than 12 Muts/Mb and lower than 100 Muts/Mb were considered hypermutated (22, 23), and patients with TMB higher than 100 Muts/Mb were recognized as ultrahypermutated (24). The chromosomal instability (CIN) score was calculated with the mean percentage of genes with abnormal (log2 ratio > ± 0.2) copy numbers, weighted on 22 autosomal chromosomes. All variant calling, MSI, CIN, and TMB definitions were CLIA/CAP accredited.

### Data analysis

Statistical analyses were performed in R (v4.2.1). The differences in numerical variables were compared using the Fisher’s exact test implemented in R. The differences between groups were compared using the Wilcoxon rank-sum test in R. Survival analyses were performed using the Kaplan-Meier method and multivariate cox analysis. P values in multiple comparisons were FDR (false discovery rate) adjusted and tests with P values or FDR less than 0.05 were considered to be statistically significant. In the gene set enrichment analysis (GSEA) (25), an FDR less than 0.25 was considered significant according to the software manual.

## Results

### Clinicopathological characteristics

**Figure 2:**
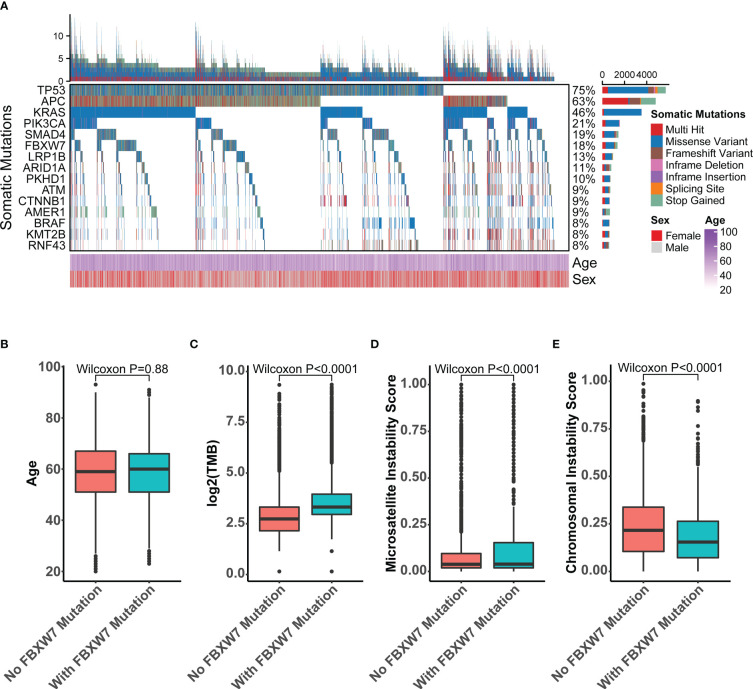
Molecular characteristics of FBXW7 somatic mutations in the recruitment cohort. **(A)** Oncoprint of top 15-mutated genes in the recruitment cohort; **(B)** Boxplot of the age of FBXW7-mutated and FBXW7-wild type patients; **(C)** Boxplot of the tumor mutation burden of FBXW7-mutated and FBXW7-wild type patients; **(D)** Boxplot of the microsatellite instability score of FBXW7-mutated and FBXW7-wild type patients; **(E)** Boxplot of the chromosomal instability scores of FBXW7-mutated and FBXW7-wild type patients.

We analyzed the sequencing data for 7626 CRC samples profiled by targeted next-generation sequencing. The clinical features of this cohort were comparable to previous CRC studies, such as 60% were male patients, a representative age distribution with a median of 59 (26) (**Table S1**). The most mutated genes in this cohort included TP53, APC, KRAS, PIK3CA, SMAD4, and FBXW7, which accounted for 75%, 63%, 46%, 21%, 19%, 18% of total nonsynonymous mutations, respectively (**Figure 2A**). A total of 1365 FBXW7 mutated patients were identified in the cohort. There were no discrepancies in age (**Figure 2B**) and gender (P = 0.879) between FBXW7 mutated and wild-type (WT) CRC patients (**Table S1**). Microsatellite (P < 0.001) and hypermutation (P < 0.001) status were significantly associated with FBXW7 mutation status (**Table S1**). FBXW7 mutated CRC patients showed higher TMB (P < 0.001, **Figure 2C**), higher MSI score (P < 0.001, **Figure 2D**), and lower CIN score (P < 0.001, **Figure 2E**). Almost all gene mutations with a minor allele frequency (MAF) larger than 5%, except SMAD4 and BRAF, were positively associated with FBXW7 mutations (FDR < 0.001, odds ratio [OR] > 1.0, **Table S3**). Only TP53 mutations were negatively associated with FBXW7 mutations (FDR < 0.001, OR = 0.749, **Table S3**).

### FBXW7 mutated patients showed better overall survival

**Figure 3:**
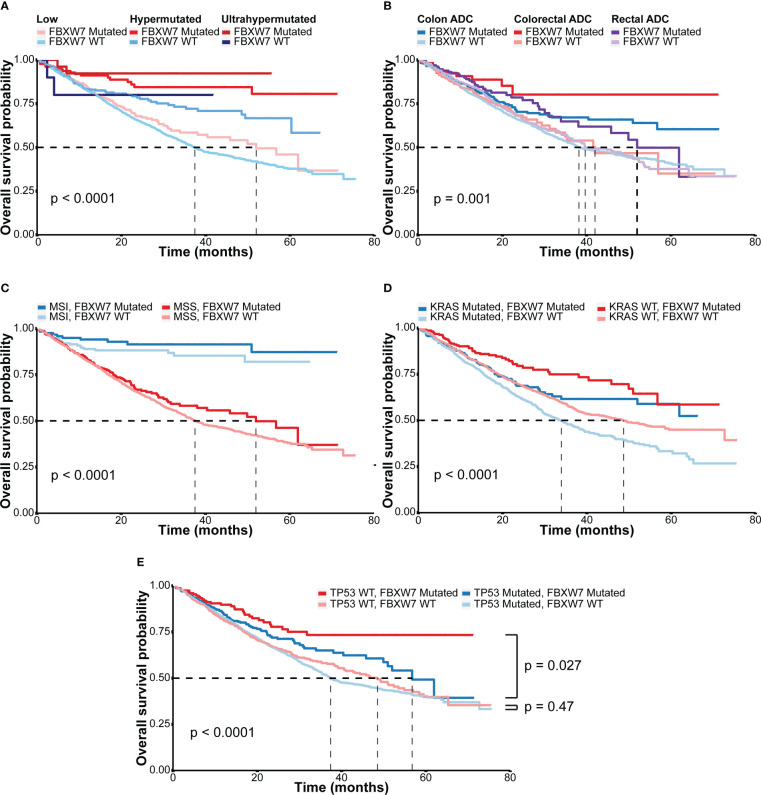
Overall survival outcomes of different subgroups in the MSK cohort. **(A)** Kaplan-Meier overall survival curves of colorectal cancer patients according to hypermutation status and FBXW7 mutation status; **(B)** Kaplan-Meier overall survival curves of colorectal cancer patients according to tumor sites and FBXW7 mutation status; **(C)** Kaplan-Meier overall survival curves of colorectal cancer patients according to microsatellite status and FBXW7 mutation status; **(D)** Kaplan-Meier overall survival curves of colorectal cancer patients according to KRAS mutation status and FBXW7 mutation status; **(E)** Kaplan-Meier overall survival curves of colorectal cancer patients according to TP53 mutation status and FBXW7 mutation status.

In the MSK MetTropism cohort, colorectal cancer patients with FBXW7 somatic mutations had better overall survival outcomes (hazard ratio [HR]: 0.67; 95% confidence interval [CI]: 0.55-0.80, P < 0.001, **Figure S1A**). Regarding the hypermutation status, for both FBXW7 mutated and WT patients, ultrahypermutated patients showed better overall survival, followed by hypermutated patients (**Figure 3A**, P < 0.001). FBXW7 mutated patients had better prognosis outcomes in all hypermutation statuses (**Figure 3A**). Similarly, the same trend was discovered regarding primary tumor locations (**Figure 3B**) and KRAS mutation status (**Figure 3D**). However, this was not confirmed in the multivariate Cox model, with covariates including MSI status, age, sex, TMB (**Figure S2**). The MSI status was the most significant factor affecting overall survival outcomes(P < 0.001, **Figure S2**). Nevertheless, FBXW7 mutated MSI and MSS patients both showed a better trend in overall survival (**Figure 3C**), though were not statistically significant (**Figures S1B, C**). Interestingly, in the FBXW7 mutated group, TP53 mutated patients had significantly worse overall survival outcomes than their WT counterpart (P = 0.027), while among FBXW7 WT patients, there was no difference in prognosis between the two groups (P = 0.47, **Figure 3E**).

### FBXW7 R465C is a negative overall survival indicator

**Figure 4:**
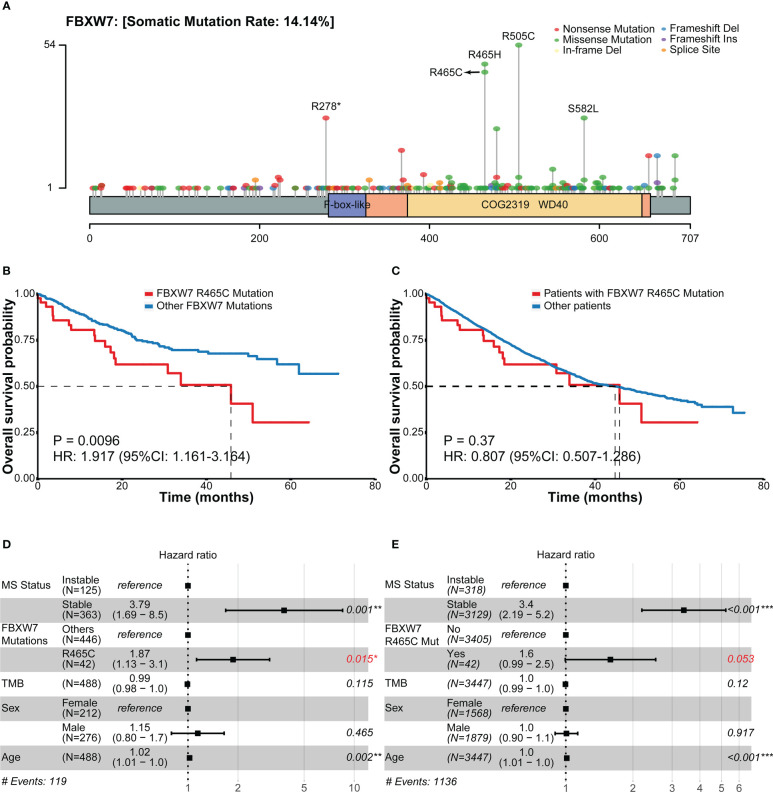
Frequencies of FBXW7 mutation sites and related overall survival outcomes in the MSK cohort. **(A)** Lollipop plot unveiling the mutation frequencies of each site of FBXW7; **(B)** Kaplan-Meier overall survival curves of between colorectal cancer patients carrying FBXW7 R465C mutations and other FBXW7 mutations; **(C)** Kaplan-Meier overall survival curves of between colorectal cancer patients carrying FBXW7 R465C mutations and not carrying FBXW7 R465C mutations; **(D)** Forest plot of overall survival regarding the MS status, FBXW7 R465C mutation status, TMB, sex, age in FBXW7-mutated patients; **(E)** Forest plot of overall survival regarding the MS status, FBXW7 R465C mutation status, TMB, sex, age in all patients. HR, hazard ratio; CI, confidential interval.

The overall survival outcomes of each site of FBXW7 mutations were explored using the MSK cohort. The most frequent mutations of FBXW7 were R505C, R465H, R465C, R278*, S582L (**Figure 4A**), and missense mutations were the most dominant mutation type. Patients carrying different FBXW7 somatic alterations showed similar overall survival outcomes, apart from those carrying R465C mutations, who had a worse prognosis compared to other FBXW7 mutated patients (HR: 1.92, 95% CI: 1.16-3.16, P = 0.0096, **Figure 4B**). In the multivariate cox analysis within FBXW7 mutated patients, the R465C alteration was still identified as a significant negative factor (HR: 1.87; 95% CI: 1.13-3.1, P = 0.015, **Figure 4D**). When compared to all other patients who did not carry FBXW7 R465C alterations, the Kaplan-Meier model did not show significant differences (HR: 0.81, 95% CI: 0.51-1.29, P = 0.37, **Figure 4C**), while in the multivariate cox analysis, after MSI status correction, the R465C alteration was identified as a negative indicator for OS (HR: 1.6; 95% CI: 0.99-2.5, P = 0.053, **Figure 4E**).

### Immune cell infiltration and gene set enrichment analysis

**Figure 5:**
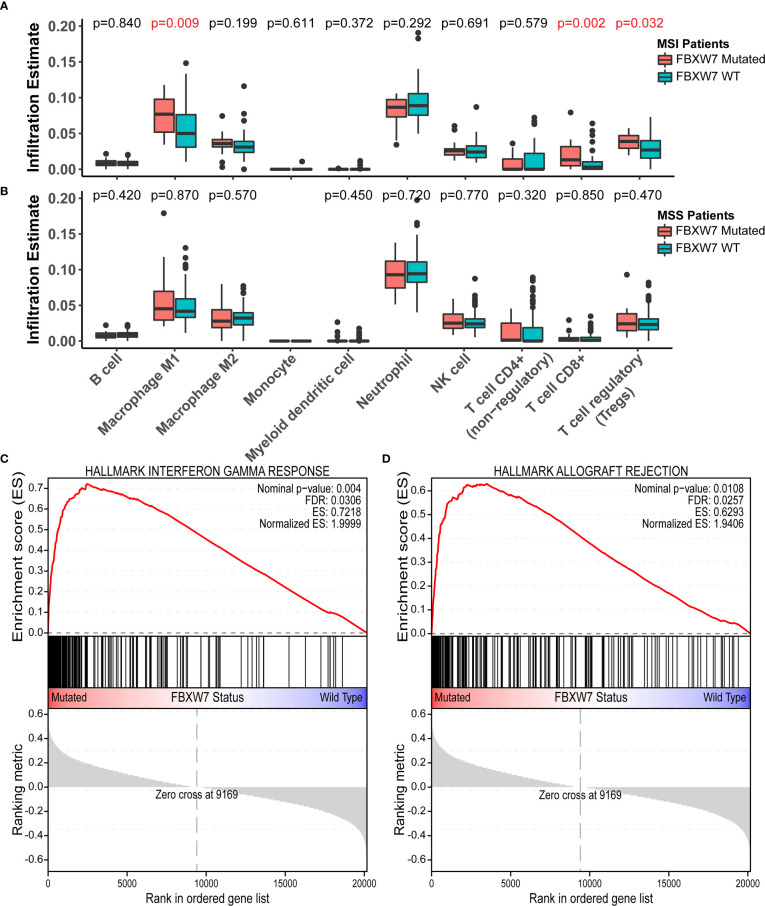
Immune cell infiltration analysis and gene-set enrichment analysis in the TCGA COAD cohort. **(A)** Boxplots of immune cell infiltration rates in microsatellite instable patients regarding different FBXW7 mutation status; **(B)** Boxplots of immune cell infiltration rates in microsatellite stable patients regarding different FBXW7 mutation status; **(C)** Hallmark gene set interferon gamma response enrichment plot in TCGA COAD patients regarding FBXW7 mutation status; **(D)** Hallmark gene set allograft rejection enrichment plot in TCGA COAD patients regarding FBXW7 mutation status.

263 patients with RNA sequencing data in the TCGA COAD cohort were used for immune cell infiltration and gene set enrichment analysis (GSEA). The cohort was divided into four groups based on their FBXW7 mutation status and MSI status and compared, as FBXW7 was previously found to be highly associated with MSI. There was no difference in the infiltration rates of all immune cells between FBXW7 mutated and WT groups in microsatellite stable patients (**Figure 5A**), while MSI FBXW7 mutated patients had higher M1 macrophage (P = 0.009), CD8+ T cell (P = 0.002), and regulatory T cell (P = 0.032) infiltration rates (**Figure 5B**). In the GSEA analysis, 11 hallmark gene sets were enriched in the FBXW7 mutated phenotype in MSI patients, including interferon-gamma response, interferon-alpha response, TNFA signaling *via* NFKB, inflammatory response, IL6 JAK STAT3 signaling, complement, p53 pathway, IL2 STAT5 signaling, apoptosis (**Figures 5C, D**; **Table S2**). No gene sets were significantly enriched in the other three groups (MSI FBXW7 WT, MSS FBXW7 mutated, MSS FBXW7 WT).

## Discussion

FBXW7 mutation rate was recorded as 2.54% throughout all human cancer, of which 72.70% were missense mutations and 13.82% were nonsense mutations (27). FBXW7 is a topic of increasing interest in cancer research, as it serves a role in protein ubiquitylation and proteosome-mediated degradation, which is responsible for the regulation of multiple crucial oncogenes. FBXW7 also plays a part in the signaling pathway regulating cell apoptosis, cell proliferation, cell cycle, tumorigenesis and invasion, DNA damage repair (DDR), genomic instability and telomere (28–30). FBXW7 had a high mutation rate in colorectal cancer patients of about 18% (**Figure 2A**) and inactivation of it may induce the disruption of the above-mentioned pathways, which may lead to tumorigenesis. However, detailed characterizations of FBXW7 in colorectal cancer patients is still lacking. In this study, we performed a comprehensive profiling of FBXW7 in CRC.

FBXW7 mutated CRC patients were found to have higher MSI scores, higher TMB, and lower CIN scores. It was reported before that FBXW7 was involved in the DNA damage response and repair by promoting the degradation of p53, PLK1, SOX9, and BLM upon DNA damage stress (29, 31–33), inactivation of which may disrupt this process and resulting in a higher TMB value. In previous studies, the majority of MSI-high patients were identified as TMB-high (34), and CRC patients with defective DNA damage response more commonly had MSI-H tumors (35), which may help explain the higher TMB and MSI scores in FBXW7 mutated patients. Though FBXW7 inactivation was considered CIN-promoting (36, 37), we observed decreased CIN scores in FBXW7 mutated CRC patients. As FBXW7 mutated patients were significantly enriched in the MSI group and had higher TMB, which means carrying more somatic mutations, it was predictable that FBXW7 mutations were positively correlate to other gene mutations.

The association between FBXW7 mutations and prognosis remains controversial among various studies. A study with 855 metastatic CRC patients reported FBXW7 missense mutation as a strong negative factor for OS (P = 0.003) (16). However, another study including 1519 CRC patients found that FBXW7 mutated patients showed a constant better trend in 5-year survival, though not statistically significant (P = 0.665) (12). Disease-free survival of FBXW7 mutated CRC patients was significantly better than that of FBXW7 WT patients in the TCGA colorectal cohort (P < 0.001) (28). Interestingly, in a meta-analysis studying 4199 CRC patients, it was stated that FBXW7 mutations were associated with advanced T stages and lymph node metastases (13). In our study, FBXW7 mutated patients had numerically longer OS, as no statistically significant differences were highlighted in the multivariate Cox analysis. However, FBXW7 mutated patients displayed continuous better OS in both MSI and MSS patients, while MSI status was the most important indicator for OS (**Figure 3C**).

p53 regulation of FBXW7 may explain the disparities in patients with different FBXW7 mutation statuses in terms of OS between TP53 mutated and WT patients (**Figure 3E**). The tumor suppressor protein p53, encoded by TP53, was reported to be responsible for promoting cell cycle arrest to allow DNA repair and/or apoptosis under cellular stress (38, 39), and inactivation of TP53 was considered as a promoter of tumorigenesis. Moreover, after DNA damage, the p53 level will decrease through FBXW7-involved protease-mediated degradation to induce cell proliferation recovery (40), while decreased p53 levels may lead to tumor survival and resistance to treatments (40). Hence, FBXW7-mutated and TP53-WT patients may have a better tumor prognosis, as the p53 degradation process was inactivated and a high p53 level was maintained.

Regarding the prognosis significance of each FBXW7 mutation sites, in a previous study, patients with FBXW7 R465H/R465C/R479Q mutations were reported to have better overall survival outcomes compared to other mutation sites (12). However, the results for single mutation sites were missing. In our study, interestingly, we discovered that patients carrying FBXW7 R465C mutation had worse OS when compared to patients carrying all other FBXW7 mutations (P = 0.0096), even when compared to patients with FBXW7 R465H of the same site (HR: 3.08, 95% CI: 1.28-7.39, P = 0.0082, **Figure S3**). It was also labeled as a negative prognosis indicator in multivariate cox analysis of all CRC patients after MSI status correction (P = 0.053). R465 is the most commonly mutated site of FBXW7 in human cancers and was considered a loss-of-function mutation (9). WT FBXW7 and FBXW7 R465C mutant co-expression was associated with increasing steady-state cyclin E1 level and half-life (41). Also, FBXW7 R465C mutants were found unable to degrade c-MYC and BRAF, which were recognized as substrates of WT FBXW7 (42). Notably, in a previous study, it is hypothesized that at low PH, FBXW7 R465H may function normally and bond to substrates stably, while at high PH, histidine is more likely to be neutral, which can cease binding to the substrates (43). However, the mechanisms of how FBXW7 R465C mutations influenced the overall survival of CRC patients were still unknown.

In the immune cell infiltration analysis, we found higher infiltration rates of M1 macrophages, CD8+ T cells, and Tregs in FBXW7 mutated MSI patients. Multiple immune-related gene sets, including interferon-gamma and alpha response, inflammatory response, IL6, IL2, p53, and apoptosis signaling pathways, were identified as enriched in the FBXW7 mutated MSI patients. Hence, we hypothesize that, under microsatellite instable conditions, FBXW7 mutated patients had elevated immune-related activities. The mechanisms behind such phenomenon need further elucidation.

In all, we performed a comprehensive profiling of FBXW7 mutation in CRC patients using three independent cohorts discovering that the FBXW7 mutations were associated with higher TMB, higher MSI scores, and lower CIN scores. We reported for the first time that FBXW7 R465C was predictive for worse OS, and the FBXW7 mutated group in MSI patients showed elevated immune-related activities compared to MSS patients. However, due to the lack of some clinicopathological information on data sets used in this study, the clinical impact of FBXW7 in colorectal cancer was not fully uncovered. Future studies with complete clinical features are warranted.

## Data availability statement

The data that support the findings of this study are available from the corresponding author upon reasonable request.

## Ethics statement

The studies involving human participants were reviewed and approved by the Ethical Committee of The First Affiliated Hospital of Nanjing Medical University (Approval No. 2022-SR-294). The patients/participants provided their written informed consent to participate in this study.

## Author contributions

Conceptualization: YL. Methodology: HB, RW, LZ. Validation: JZ. Formal analysis: YL, HC, HB. Data curation: JZ. Supervision: RW, LZ. Original draft preparation: YL, HC, HB, JZ. Review and editing: RW, LZ, HC, HB. All authors contributed to the article and approved the submitted version.
