# Supplementary material for: Comprehensive characterization of FBXW7 mutational and clinicopathological profiles in human colorectal cancers
Source: Front Oncol. 2023 Mar 29;13:1154432. doi: 10.3389/fonc.2023.1154432 (PMC10091464; doi:10.3389/fonc.2023.1154432)
Supplement: Supplementary file 1 [file DataSheet_1.docx]

**Table S1**

**Clinical characteristics of FBXW7 mutated and wild type colorectal cancer patients**

| **Clinical Characteristics** | **FBXW7 Mutated** | **FBXW7 WT*** | **P Values**** |
| --- | --- | --- | --- |
|  | **(n, frequency [%])** | **(n, frequency [%])** |  |
| **Sex** |  |  | 0.886 |
| Female | 537 (7.04%) | 2479 (32.51%) |  |
| Male | 828 (10.86%) | 3782 (49.59%) |  |
| **Age** |  |  | 0.880 |
| Mean | 58.26 | 58.12 |  |
| SD* | 12.24 | 12.35 |  |
| Median | 60 | 59 |  |
| **Microsatellite Status** |  |  | <0.001 |
| MSI* | 201 (2.64%) | 347 (4.55%) |  |
| MSS* | 1135 (14.88%) | 5759 (75.52%) |  |
| Unknown*** | 29 (0.38%) | 155 (2.03%) |  |
| **Hypermutation Status** |  |  | <0.001 |
| Ultrahypermutated | 119 (1.56%) | 68 (0.89%) |  |
| Hypermutated | 420 (5.51%) | 807 (10.58%) |  |
| Low | 826 (10.83%) | 5386 (70.63%) |  |

*: WT wild type, SD standard deviation, MSI microsatellite instable, MSS microsatellite stable.

**:P-values were calculated using chi-squared test, except the Age section was calculated using the Wilcoxon rank-sum test.

***: Patients with unknown microsatellite status were not included in the P-value calculation.

**Table S2**

**Gene sets enriched in phenotype FBXW7 mutated (18 samples) in MSI patients**

| **NAME** | **ES*** | **NES*** | **P value** | **FDR**** |
| --- | --- | --- | --- | --- |
| Hallmark interferon gamma response | 0.722 | 2.000 | 0.004 | 0.031 |
| Hallmark allograft rejection | 0.629 | 1.941 | 0.011 | 0.026 |
| Hallmark interferon alpha response | 0.726 | 1.829 | 0.014 | 0.060 |
| Hallmark TNFA signaling via NFKB | 0.529 | 1.648 | 0.048 | 0.172 |
| Hallmark inflammatory response | 0.508 | 1.614 | 0.055 | 0.172 |
| Hallmark IL6 JAK STAT3 signaling | 0.503 | 1.527 | 0.090 | 0.237 |
| Hallmark complement | 0.411 | 1.527 | 0.054 | 0.204 |
| Hallmark p53 pathway | 0.349 | 1.488 | 0.038 | 0.219 |
| Hallmark IL2 STAT5 signaling | 0.358 | 1.471 | 0.078 | 0.208 |
| Hallmark hypoxia | 0.369 | 1.439 | 0.087 | 0.218 |
| Hallmark apoptosis | 0.356 | 1.399 | 0.083 | 0.237 |
| Hallmark heme metabolism | 0.266 | 1.216 | 0.157 | 0.458 |
| Hallmark glycolysis | 0.280 | 1.209 | 0.197 | 0.434 |
| Hallmark apical surface | 0.317 | 1.115 | 0.313 | 0.564 |
| Hallmark PI3K AKT MTOR signaling | 0.243 | 1.085 | 0.335 | 0.577 |
| Hallmark UV response up | 0.237 | 1.022 | 0.402 | 0.654 |
| Hallmark KRAS signaling up | 0.258 | 0.988 | 0.464 | 0.680 |
| Hallmark reactive oxygen species pathway | 0.285 | 0.958 | 0.497 | 0.699 |
| Hallmark fatty acid metabolism | 0.227 | 0.917 | 0.532 | 0.745 |
| Hallmark estrogen response late | 0.195 | 0.883 | 0.684 | 0.775 |
| Hallmark mitotic spindle | 0.197 | 0.772 | 0.765 | 0.945 |
| Hallmark adipogenesis | 0.169 | 0.721 | 0.895 | 0.984 |
| Hallmark androgen response | 0.202 | 0.715 | 0.871 | 0.951 |
| Hallmark oxidative phosphorylation | 0.211 | 0.653 | 0.790 | 0.987 |
| Hallmark G2M checkpoint | 0.181 | 0.599 | 0.836 | 0.996 |
| Hallmark TGF beta signaling | 0.168 | 0.568 | 0.969 | 0.978 |
| Hallmark E2F targets | 0.158 | 0.469 | 0.929 | 0.980 |

*: ES enrichment score, NES normalized enrichment score.

**: FDR < 0.25 was considered significant according to the package manual.

**Table S3**

**Associations of gene mutations (minor allele frequency larger than 5%) with the FBXW7 mutations. FDR false discovery rate, Conf confidence interval.**

|  | **Odds ratio** | **Conf 2.5** | **Conf 97.5** | **P value** | **FDR** | **Frequency** |
| --- | --- | --- | --- | --- | --- | --- |
| **TP53** | 0.749 | 0.657 | 0.856 | 1.87E-05 | 1.97E-05 | 0.750 |
| **APC** | 2.343 | 2.040 | 2.697 | 2.09E-37 | 2.04E-36 | 0.630 |
| **KRAS** | 1.610 | 1.429 | 1.816 | 2.24E-15 | 3.01E-15 | 0.464 |
| **PIK3CA** | 1.794 | 1.567 | 2.052 | 3.78E-17 | 6.14E-17 | 0.207 |
| **SMAD4** | 1.049 | 0.901 | 1.218 | 0.542625 | 0.543 | 0.190 |
| **LRP1B** | 2.039 | 1.742 | 2.384 | 2.30E-18 | 3.90E-18 | 0.127 |
| **ARID1A** | 2.334 | 1.977 | 2.751 | 7.74E-23 | 1.59E-22 | 0.106 |
| **PKHD1** | 2.675 | 2.256 | 3.166 | 1.34E-28 | 6.54E-28 | 0.096 |
| **ATM** | 2.566 | 2.162 | 3.041 | 4.01E-26 | 1.20E-25 | 0.095 |
| **CTNNB1** | 1.541 | 1.274 | 1.857 | 7.63E-06 | 8.27E-06 | 0.091 |
| **AMER1** | 2.096 | 1.749 | 2.507 | 2.98E-15 | 3.88E-15 | 0.089 |
| **BRAF** | 1.138 | 0.921 | 1.397 | 0.219 | 0.224 | 0.085 |
| **KMT2B** | 3.261 | 2.737 | 3.881 | 3.30E-38 | 6.44E-37 | 0.085 |
| **RNF43** | 1.698 | 1.397 | 2.056 | 1.21E-07 | 1.35E-07 | 0.083 |
| **FAT1** | 3.560 | 2.975 | 4.255 | 6.09E-42 | 2.38E-40 | 0.079 |
| **GNAS** | 2.181 | 1.802 | 2.631 | 3.24E-15 | 4.08E-15 | 0.078 |
| **BRCA2** | 3.288 | 2.721 | 3.968 | 2.17E-33 | 1.69E-32 | 0.071 |
| **ERBB4** | 2.240 | 1.82 | 2.739 | 2.87E-14 | 3.50E-14 | 0.066 |
| **GRIN2A** | 3.149 | 2.584 | 3.830 | 9.72E-29 | 5.41E-28 | 0.065 |
| **NF1** | 3.082 | 2.526 | 3.754 | 2.13E-27 | 6.93E-27 | 0.064 |
| **CREBBP** | 3.737 | 3.068 | 4.546 | 1.62E-37 | 2.04E-36 | 0.063 |
| **ALK** | 3.025 | 2.469 | 3.699 | 1.31E-25 | 3.66E-25 | 0.061 |
| **TGFBR2** | 2.830 | 2.300 | 3.475 | 5.13E-22 | 1.00E-21 | 0.060 |
| **KMT2A** | 3.218 | 2.622 | 3.941 | 8.08E-28 | 3.15E-27 | 0.060 |
| **NOTCH1** | 2.419 | 1.957 | 2.981 | 9.52E-16 | 1.38E-15 | 0.060 |
| **MTOR** | 2.774 | 2.248 | 3.416 | 1.33E-20 | 2.47E-20 | 0.058 |
| **AXIN2** | 2.186 | 1.748 | 2.721 | 1.34E-11 | 1.53E-11 | 0.055 |
| **CHD4** | 3.171 | 2.563 | 3.916 | 2.29E-25 | 5.58E-25 | 0.055 |
| **PTEN** | 2.815 | 2.26 | 3.488 | 5.46E-20 | 9.67E-20 | 0.054 |
| **ARID2** | 3.205 | 2.586 | 3.964 | 1.80E-25 | 4.69E-25 | 0.054 |
| **ERBB3** | 2.565 | 2.055 | 3.192 | 2.92E-16 | 4.39E-16 | 0.053 |
| **EPHA3** | 2.606 | 2.088 | 3.242 | 8.25E-17 | 1.29E-16 | 0.053 |
| **FLT4** | 3.378 | 2.723 | 4.181 | 1.80E-27 | 6.38E-27 | 0.053 |
| **PREX2** | 2.316 | 1.846 | 2.894 | 8.11E-13 | 9.58E-13 | 0.052 |
| **SMARCA4** | 2.533 | 2.025 | 3.158 | 1.08E-15 | 1.51E-15 | 0.052 |
| **ATR** | 3.112 | 2.502 | 3.862 | 1.48E-23 | 3.38E-23 | 0.052 |
| **ROS1** | 3.459 | 2.785 | 4.289 | 4.16E-28 | 1.80E-27 | 0.052 |
| **PIK3R1** | 3.137 | 2.516 | 3.903 | 2.78E-23 | 6.03E-23 | 0.051 |
| **POLE** | 3.871 | 3.117 | 4.802 | 3.15E-33 | 2.05E-32 | 0.051 |


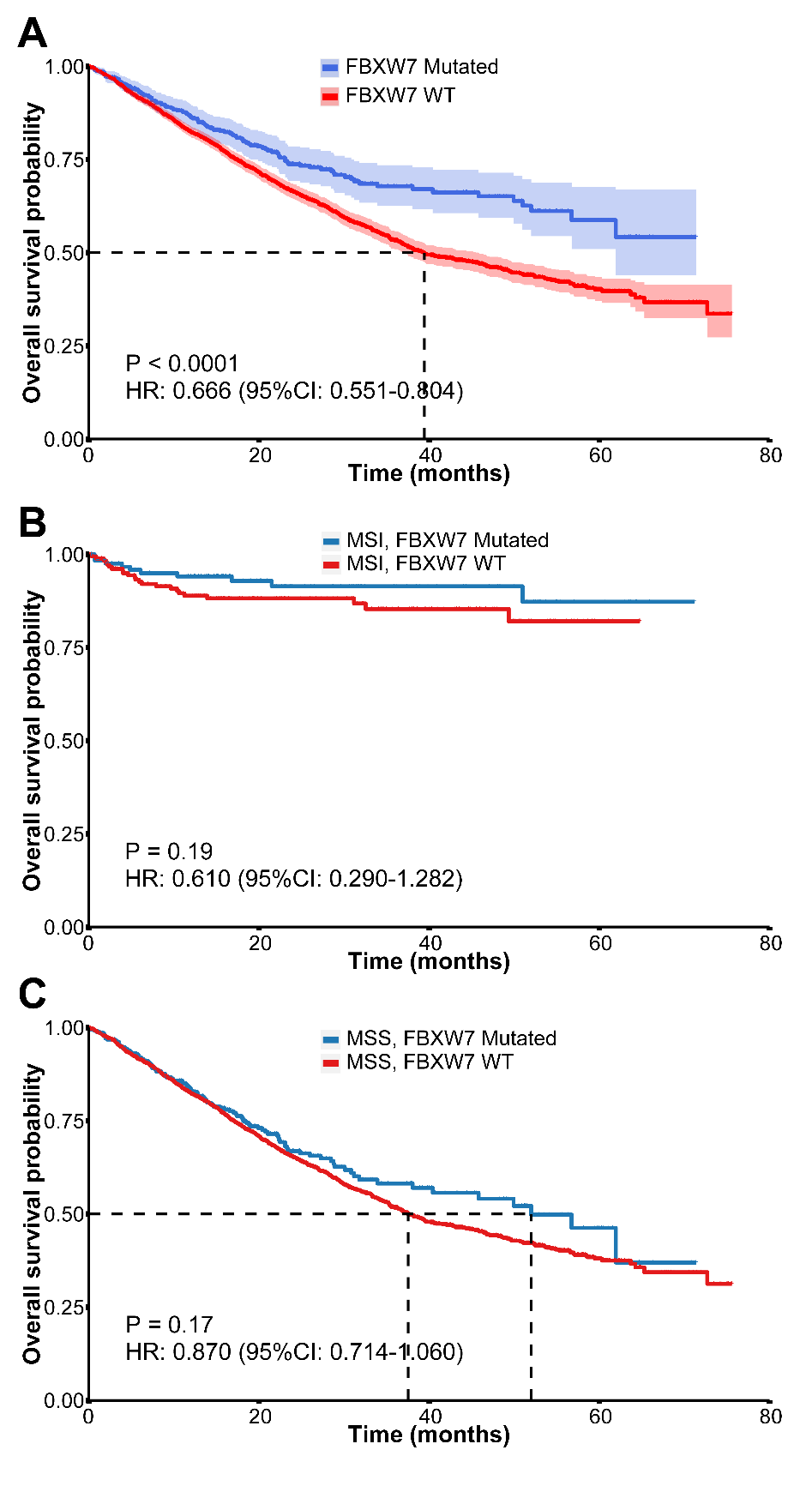


**Figure S1: Kaplan–Meier curves of overall survival between FBXW7 mutated and FBXW7 wild type in (A) all colorectal cancer patients, (B) microsatellite instable patients, and (C) microsatellite stable patients.**

**Note: HR hazard ratio, CI confidential interval.**


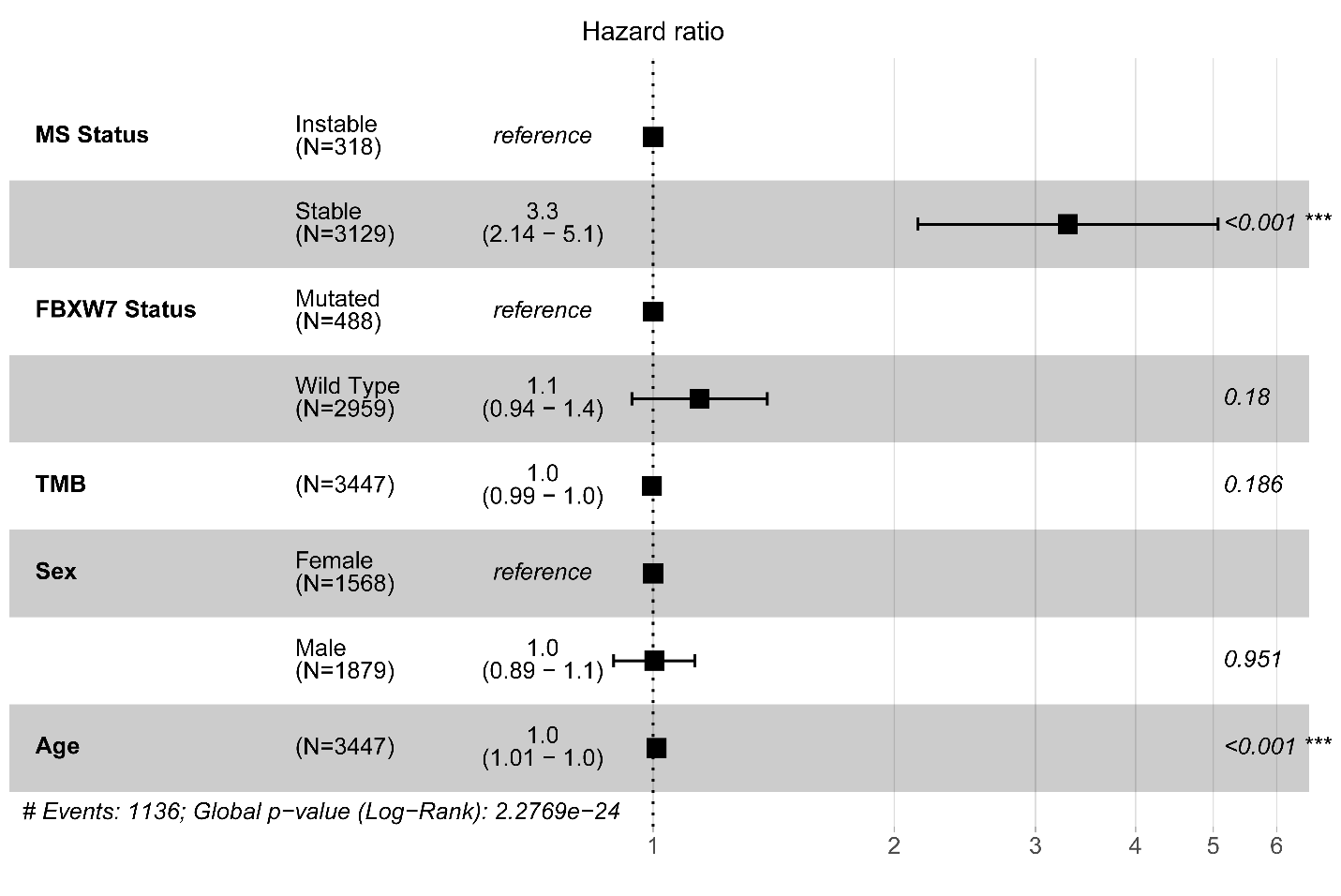


**Figure S2: Forest plot of overall survival in the MSK MetTropism cohort.**


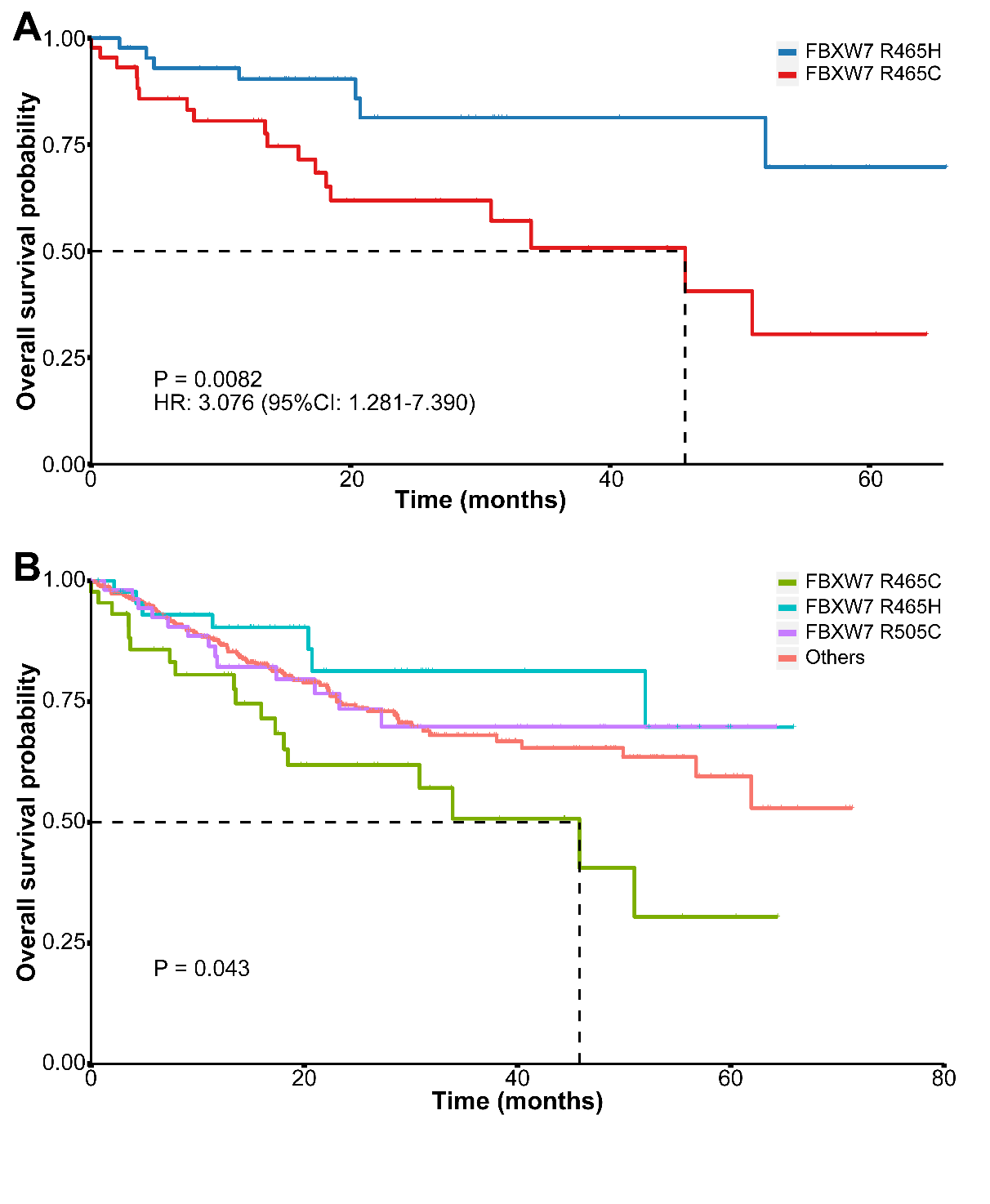


**Figure S3: Kaplan–Meier curves of overall survival (A) of FBXW7 R465H and FBXW7 R465C mutated colorectal cancer patients, (B) FBXW7 R505C, FBXW7 R465H, and FBXW7 R465C mutated colorectal cancer patients.**

**Note: HR hazard ratio, CI confidential interval.**
